# Effects of Electroacupuncture for Knee Osteoarthritis: A Systematic Review and Meta-Analysis

**DOI:** 10.1155/2016/3485875

**Published:** 2016-10-13

**Authors:** Jae-Woo Shim, Jae-Young Jung, Sung-Soo Kim

**Affiliations:** ^1^Department of Clinical Korean Medicine, Graduate School, Kyung Hee University, Seoul, Republic of Korea; ^2^Hospital of Korean Medicine, Kyung Hee University Medical Center, Seoul, Republic of Korea

## Abstract

*Purpose.* This study aims to verify the effects of electroacupuncture treatment on osteoarthritis of the knee.* Methods.* MEDLINE/PubMed, EMBASE, CENTRAL, AMED, CNKI, and five Korean databases were searched by predefined search strategies to screen eligible randomized controlled studies meeting established criteria. Any risk of bias in the included studies was assessed with the Cochrane Collaboration's tool. Meta-analysis was conducted using RevMan version 5.3 software.* Results.* Thirty-one randomized controlled studies of 3,187 participants were included in this systematic review. Meta-analysis was conducted with eight studies including a total of 1,220 participants. The electroacupuncture treatment group showed more significant improvement in pain due to knee osteoarthritis than the control group (SMD −1.86, 95% CI −2.33 to −1.39, *I* ^2^ 75%) and in total WOMAC score than the control group (SMD −1.34, CI 95% −1.85 to −0.83, *I* ^2^ 73%). Compared to the control group, the electroacupuncture treatment group showed more significant improvement on the quality of life scale.* Conclusion.* Electroacupuncture treatment can relieve the pain of osteoarthritis of the knees and improve comprehensive aspects of knee osteoarthritis and the quality of life of patients with knee osteoarthritis.

## 1. Introduction

Osteoarthritis (OA) is one of the most common joint diseases [1, 2] in old people. The disease is a burden [3] on both an individual and a socioeconomic level based on the fact that 49% of the population aged 65 years and older suffers from OA in knee joints or hip joints [4]. Osteoarthritis is a degenerative disease that progressively causes degeneration in the tendons and cartilages surrounding OA-invasive joints, loss of cartilage, structural changes in subchondral bones, and osteosclerosis, with occurrence of osteophytes. Synovitis can occur when the condition is aggravated [5]. The diagnosis of knee OA in many cases relies on knee OA criteria [6] defined by the American College of Rheumatology (ACR) together with radiographic criteria depending on structural changes in joints [7].

Recent guidelines on the treatment of knee OA [8, 9] recommend conservative nonpharmacologic management; however, this is not consistent with the reality of clinical treatments [10]. Pharmaceutical treatments are temporary methods to reduce symptoms of knee OA such as analgesics (e.g., acetaminophen), nonsteroidal anti-inflammatory drugs, glucocorticoids, topical analgesics, and cartilage-protective agents [11]. In the case of unsuccessful pharmacological treatment, total knee arthroplasty is recommended as a last resort [10, 12].

Acupuncture therapy is excellent in terms of pain relief [13], affordability [14, 15], and safety [16] and is applied for different types of musculoskeletal pain disorders. Acupuncture is conditionally recommended according to the ACR guidelines on knee OA [8], and some systematic review studies [17, 18] verified the effects of acupuncture on pain control and functional recovery in knee OA patients. Although a number of studies [17–20] suggest significant effects of acupuncture therapy for OA, the effects remain controversial. The results of another systematic review study [16] and suggested guidelines [9] are inconclusive, with limitations due to uncertain results, data, and heterogeneity.

Many studies published to date on the effects of acupuncture therapy use the term acupuncture to describe a blend of both manual acupuncture (MA) and electroacupuncture (EA) [21]. According to the results of one study [22] on EA, which is the application of electrical stimulation to acupuncture techniques, EA displays greater analgesic effects for different types of pain in comparison to MA. One publication argues that EA and MA treatments are not interchangeable and thus must be separately identified for accurate study [21]. In systematic review studies, the blending of MA and EA is detrimental to the homogeneity of studies on acupuncture effects [21]. Also, a recent Cochrane review [16] revealed that EA displays more statistically significant analgesic effects for knee OA than MA. In one recent systematic review study [19], randomized controlled trials (RCTs) involving the application of MA as an experimental intervention for knee OA were reviewed with the exclusion of EA. To our knowledge, there are no systematic review studies on the effects of EA treatments on knee OA. Therefore, we conducted a systematic review study exclusively for the effects of EA on knee OA.

## 2. Objectives

The purpose of this review is to assess the clinical effects of EA on knee OA as contrasted with sham treatment, MA, or usual care such as drug therapy or physiotherapy. Study design is restricted to RCTs. The primary objective is to highlight the effects of EA on pain, which is the main symptom of knee OA. The secondary objective is to reveal the effects of EA on comprehensive evaluation of knee OA and degradation of the quality of life of patients with OA of the knee. Comprehensive evaluation of knee OA means total evaluation of the united score of knee OA symptoms and dysfunction in knee joints.

## 3. Methods

This review was conducted according to published protocol [23] (registration number: CRD42015026446), with reference to a checklist [24] of reporting guidelines in the Preferred Reporting Items for Systematic Reviews and Meta-Analyses (PRISMA).

### 3.1. Criteria for Considering Studies for This Review

#### 3.1.1. Types of Studies

This review included prospective RCTs regarding the effects of EA on patients with knee OA but excluded any nonrandomized controlled studies. No language limitation was used.

#### 3.1.2. Types of Participants

Studies on patients with OA of knee joints were included. Other studies on groups of participants with OA in other joints or with rheumatoid arthritis were included only when the data on groups of participants with knee OA were independently extracted. Any studies on participants suspected of having symptoms of knee OA but in whom the disease was not actually diagnosed were excluded. Other studies on groups of participants with complications that may affect symptoms of knee OA were also excluded. Further excluded were several studies that were conducted on limited groups of participants with certain types of knee OA diagnosed through syndrome differentiation.

#### 3.1.3. Types of Interventions

This study defined pharmacological treatments, physiotherapy, and patient education as standards of “usual care” in therapeutic approaches to knee OA in actual clinical practice. Pharmacological treatments include oral medications such as analgesics or NSAIDs, as well as medications of external application, whereas physiotherapy includes skin thermal stimulation treatment and treatment involving physical exercise.

*Experimental Group Intervention.* For the experimental group, intervention included any study that applied any form of electric stimulation to invasive acupuncture for treating knee OA. In contrast, studies involving electric application using noninvasive types of acupuncture as apparatus that is attachable and contactable to skin were excluded. One study using MA with no electrical stimulation was excluded. Any study using different acupuncture points for EA among participants according to individual diagnosis was excluded. “Usual care” was the only additive intervention that could be used with EA as an intervention for the experimental group.

*Control Group Intervention.* For control groups, included were studies that defined groups of patients experiencing MA treatment with no electrical stimulation, sham EA, sham electrical stimulation, usual care, no treatment, or patients on a waiting list for treatment. Additionally, MA with usual care, sham EA with usual care, and sham electrical stimulation with usual care were allowed as control group interventions.

#### 3.1.4. Types of Outcome Measures

*Primary Outcomes.* Primary outcomes included all indicators for evaluating pain (e.g., Western Ontario and McMaster Osteoarthritis Index (WOMAC) pain scores, the visual analogue scale (VAS), and the numerical rating scale (NRS)).

*Secondary Outcomes.* Secondary outcomes included comprehensive indicators for evaluating symptoms and functions of knee OA as a whole (e.g., indicators for WOMAC total scores and Lequesne's index) and patient quality of life (QOL) such as the Euro-QoL instrument (EQ5D) and the 36-Item Short-Form Health Survey (SF-36).

### 3.2. Search Methods for Identification of Studies

The search for relevant literature was conducted among all articles published from the dates provided in the databases and journal publications to September 2015. Databases involved in our search were MEDLINE/PubMed, EMBASE, Cochrane Central Register of Controlled Trials (CENTRAL), Allied and Complementary Medicine Database (AMED), China National Knowledge Infrastructure (CNKI), the Chinese Medicine Database, and five Korean databases of KoreaMed, Korean Medical Database (KMBASE), Korean Studies Information Service System (KISS), the National Discovery for Science Leaders (NDSL), and Oriental Medicine Advanced Searching Integrated System (OASIS). Search strategies for the MEDLINE/PubMed database are presented in Appendix. For each retrieved article, bibliographies were scanned to conduct additional searches. In cases of data that could not be searched online (e.g., hard copy), the literature was hand-searched. No search limitations were imposed in terms of year of publication or status of publication, and clinical trial registers (e.g., ClinicalTrials.gov) were also searched for ongoing or unpublished trials.

### 3.3. Data Collection and Analysis

#### 3.3.1. Selection of Studies

According to predetermined search strategies, two reviewers (JS and JJ) independently pursued literature searches among the databases above. For database articles, ambiguous literature, and hand-searched hard copies of research, the reviewers performed primary screening with the application of predetermined criteria for inclusion and exclusion after separately reading titles and abstracts. The full text of any literature that passed initial screening was read individually by the two reviewers. Again, the predetermined criteria for inclusion and exclusion were applied to the full text of studies to finally select RCTs for our systematic review. In the processes of primary screening and final selection, the reviewers reached agreement on disputed items by way of discussion and consulted with a third reviewer (SK) on final decisions about whether to include ambiguous items in the systematic review.

#### 3.3.2. Data Extraction and Management

The reviewers extracted information from each article in accordance with a standardized form through a full-text review of the finally selected articles. Extracted information comprised demographic data about participant groups, standards of diagnosis, sample sizes in full research articles, intervention types for experimental groups, times of treatment implemented, duration periods of full treatment, numbers of participants in experimental groups, intervention types for control groups, numbers of participants in control groups, scales for outcome measurement, evaluation time points, acupuncture points on which an intervention and electrical stimulation were implemented, frequency of EA, and duration of EA. The reviewers made final decisions on any items about which they could not agree following consultation with an arbiter, SK.

#### 3.3.3. Risk of Bias Assessment

To assess risk of bias in each of the finally selected articles, the Cochrane risk of bias (ROB) tool [25] was used as a type of checklist. The reviewers assessed each of the seven domains of random sequence generation, allocation concealment, blinding of participants, personnel, outcome assessment, incomplete outcome data, selective reporting, and other biases in order to determine the level of potential risk of bias from options including high risk of bias, low risk of bias, or unclear risk of bias. For items on which the reviewers were not able to agree, consultation with the arbiter (SK) was used to reach final decisions.

#### 3.3.4. Quantitative Data Synthesis

Meta-analysis of continuous data was implemented. A weighted mean difference (WMD) was employed when the same scale was used, whereas a standardized mean difference (SMD) was employed when different scales for the same outcome were used.

In cases of studies with a crossover research design, data from first sessions preceding the crossover were obtained. When mixed data (findings from before and after a crossover) were encountered, raw data preceding the crossover were requested from the corresponding authors of the original articles. For other missing data, we contacted corresponding authors to request the missing data or any other available data.

With considerable heterogeneity between studies, a random effects model (which provides more conservative estimates of the significance of treatment effects) was employed to pool data. Therefore we used the random effects model to pool data for all meta-analyses. Meta-analysis was conducted using Cochrane Collaboration software (Review Manager Software Version 5.3). When the value of *I* ^2^ in Cochrane's Higgins *I* ^2^ statistic was greater than 75%, considerable heterogeneity was identified. Accordingly, data was not pooled. A subgroup analysis was conducted in order to identify reasons for heterogeneity when the results were determined to be considerably heterogeneous.

In conducting meta-analysis based on the guidelines of prior research [16], the proper observation duration was determined after taking into account the actual clinical period required to observe effectiveness in acupuncture treatments for chronic diseases, including knee OA, along with any heterogeneity found between periods of treatment and periods of assessment in a given research. Therefore, most meta-analyses in this article were conducted on research data from observation periods longer than five weeks. If assessing outcomes for evaluating the comprehensive indicators of knee OA or pain intensity of knee OA in articles employ more than two scales, WOMAC score (used most commonly for knee OA assessment in the RCTs herein) was preferentially used.

#### 3.3.5. Subgroup Analysis

Due to the variety of interventions applied in the control group, the group was analyzed by subgroup. Subgroups received sham EA treatment, MA treatment, or pharmacological treatment depending on the types of interventions in the control group.

## 4. Results

### 4.1. Characteristics of Studies

**Figure 1:**
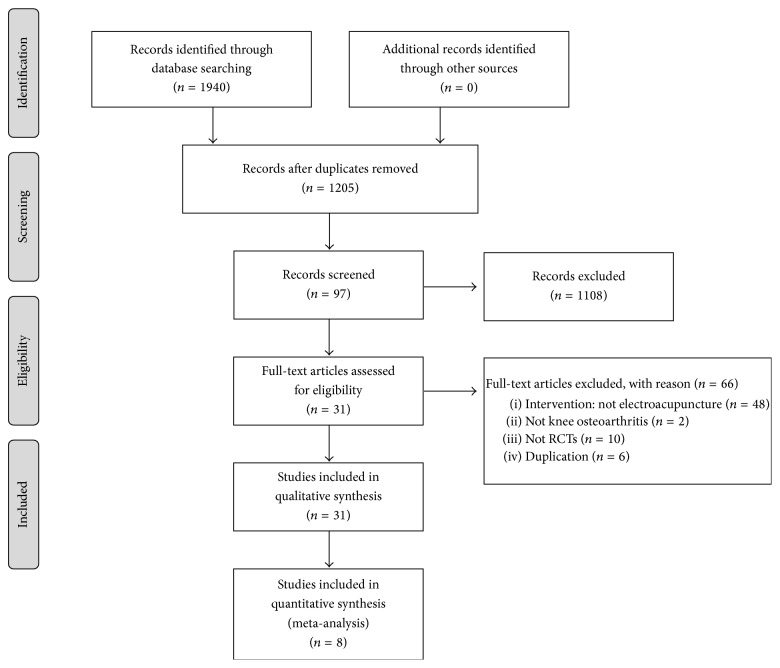
Flow diagram of the study selection process.

A total of 1,940 articles were retrieved by manual and online searches. Of these, 1,909 articles were excluded, and 31 RCTs with a total of 3,187 registered participants were finally included. Reasons for exclusion and the selection flow are presented in Figure 1.  Table 1 presents important data from the 31 included RCTs. The included RCTs were published between 1999 and 2015, with 13 of them published in English [26–38] and 18 of them published in Chinese [39–42, 42–56]. The included studies were implemented in various countries, including one study each from Brazil, Greece, Pakistan, Thailand, Turkey, and Spain. Two publications from Hong Kong [28, 35], two from the United Kingdom [31, 33], and two from the United States of America [32, 37] were included, as well as 19 publications from China [29, 39–56].

### 4.2. Characteristics of Interventions Used in Experimental and Control Groups

Periods of EA treatments for experimental groups ranged from one day to 26 weeks (Table 1). There were 20 studies [27, 31–34, 36, 37, 39, 40, 42–49, 52, 53, 55] involving more than four weeks of EA treatments for patients, while 11 studies [26, 28–30, 35, 38, 41, 50, 51, 54, 56] involved fewer than four weeks of EA treatments for patients.

Data about interventions of experimental and control groups are presented in Table 1. For the experimental groups, 23 studies [26, 29–32, 35, 37–39, 43–56] exclusively used EA as an intervention, and eight other studies [27, 28, 33, 34, 36, 40–42] used both EA and drug therapies (with three of these allowing participants to reduce medication dosages depending on symptoms). Even in the 23 studies applying only EA interventions for experimental groups, a painkiller was administered in most cases of severe pain. In most studies, the original medications of participants were maintained. There were seven studies [26, 39, 41, 43, 46–48] that used MA as an intervention in control groups, while six studies [27, 29–32, 34] used sham EA as an intervention in control groups. The remaining two studies [27, 34] used both sham EA and drug therapies in control groups. There were two studies [36, 38] that used sham electrical stimulation as an intervention in control groups, and one of these [36] used both sham electrical stimulation and medication treatments. There were 12 studies [33, 37, 40, 42–45, 49–56] that used drug therapy as the only intervention in control groups, in contrast to one study [28] that used both drug therapy and physiotherapy for interventions in the control group. Another single study [35] used general patient education as an intervention for the control group.

In most studies, participants in experimental groups used the same acupuncture points for treatments, whereas three studies [34, 43, 52] applied MA with the addition of individualized points depending on the diagnosis of participants. The frequency of electrical stimulation was between 2 hertz (Hz) and 100 Hz and was applied for a range of time between 20 and 60 minutes. Details of EA treatments are displayed in Table 2, referring to the Standards for Reporting Interventions in Clinical Trials of Acupuncture (STRICA) [57].

### 4.3. Risk of Bias in Included Studies

**Figure 2:**
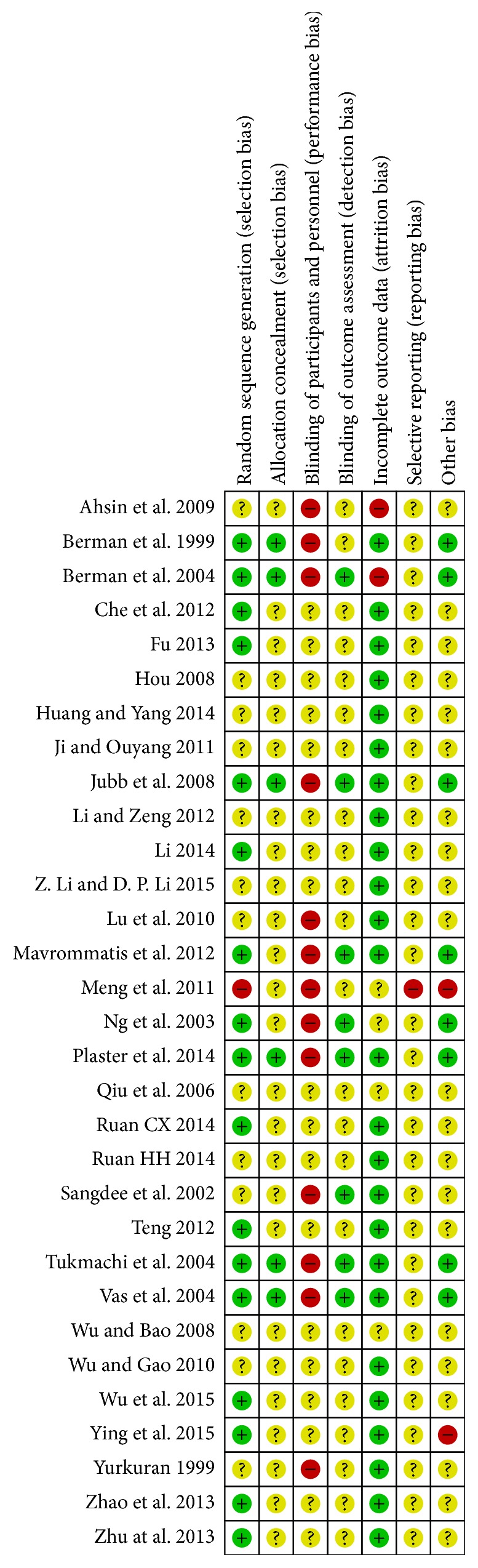
Assessment of risk of bias.

Of the studies included herein, 17 [26, 27, 31–35, 37, 39, 41, 43–45, 47, 50, 54, 55] used the proper randomization method, and one article [28] failed to use the proper randomization method. The remaining 13 articles did not provide specific descriptions about their randomization methods (Figure 2). Six studies [26, 31–34, 37] adequately carried out allocation concealment, and the other studies did not describe in detail their processes of allocation concealment. No studies were evaluated as low risk in terms of the characteristics of EA with acupuncture according to a double-blind design for the assessment of participants and personnel blinding. Thirteen studies [26–37] in which participants were single-blinded were evaluated as high risk, and the other studies did not mention whether or not they carried out methods for participant blinding. Eight studies [26, 27, 31–36] provided clarification of blinding methodologies on assessment of outcomes, but this clarification did not necessarily impact the results of the research given the fact that most outcomes were based on subjective questionnaires such as the VAS. Two studies [30, 32] showed a significant dropout rate in the domain of incomplete outcome data, and four other studies [28, 35, 49, 53] did not mention dropout or withdrawal.

### 4.4. Effects of Interventions

Eight studies [27, 31–34, 37, 43, 45] with a total of 1,220 participants were included in our meta-analysis. Meta-analysis was conducted using data observed over a period of more than five weeks, except the mental state-related QOL scales. The SF-36 physical scale was used exclusively for physical state-related QOL outcomes, whereas WOMAC total scale scores were used exclusively to evaluate comprehensive outcomes of knee OA. The remaining meta-analyses were conducted on the remaining outcomes by synthesizing multiple scales. Three articles [32, 40, 41] reported data that evaluated outcomes after more than 24 weeks of treatment, and other articles reported data that evaluated a range of treatment periods lasting longer than five weeks and shorter than 14 weeks. A previous study [16] conducted meta-analyses separately according to the evaluation time points (short- and longer-term time points). We concluded that there was a considerable heterogeneity between less than 14 weeks and more than 24 weeks of outcome data. Therefore, we conducted meta-analysis separately according to the evaluation time points (less than 14 and more than 24 weeks) like the previous study [16]. However the meta-analysis of outcome data at more than 24 weeks was not suggested because of the substantial heterogeneity.

We based one meta-analysis (Figure 5) on change scores, which indicate the amount of change from baseline to final measurement points. Other meta-analyses were conducted using final scores of final measurement points.

#### 4.4.1. Primary Outcomes

**Figure 3:**
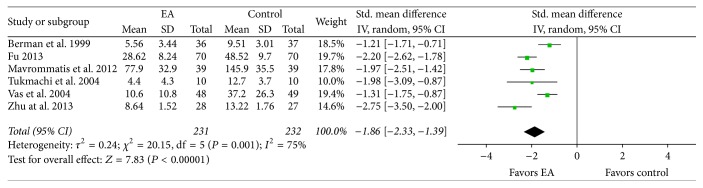
Effects of EA treatment versus control interventions on pain intensity. EA: electroacupuncture.

**Figure 4:**
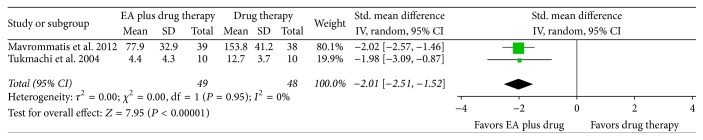
Effects of EA treatment plus drug therapy versus drug therapy alone on pain intensity. EA: electroacupuncture.

**Figure 5:**
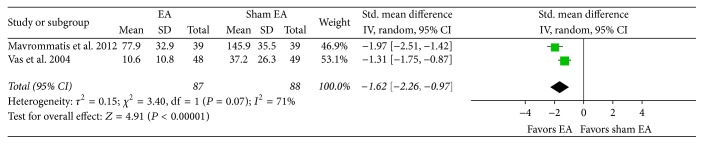
Effects of EA treatment versus sham EA on pain intensity. EA: electroacupuncture.

**Figure 6:**
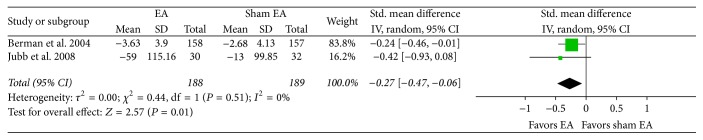
Effects of EA treatment versus sham EA on pain intensity (meta-analysis using change scores from baseline). EA: electroacupuncture.

In the meta-analysis of six eligible studies [27, 33, 34, 37, 43, 45] in which 463 subjects participated, the EA treatment group showed more significant improvement in pain due to knee OA than the control group (SMD −1.86, 95% CI −2.33 to −1.39, and *I* ^2^ 75%; Figure 3). As prearranged, a subgroup analysis was conducted according to control group interventions. The EA treatment plus drug therapy group showed more significant improvement in pain due to knee OA than the group receiving drug therapy treatment (SMD −2.01, CI 95% −2.51 to −1.52, and *I* ^2^ 0%; Figure 4). Also, the EA treatment group showed more significant improvement in pain due to knee OA than the group receiving sham EA treatment (SMD −1.62, CI 95% −2.26 to −0.97, and *I* ^2^ 71%; Figure 5). In the meta-analysis using change score data (indicating the amount of change from baseline values), the EA treatment group showed more significant improvement in pain due to knee OA than the sham EA group (SMD −0.27, CI 95% −0.47 to −0.06, and *I* ^2^ 0%; Figure 6).

#### 4.4.2. Secondary Outcomes

**Figure 7:**
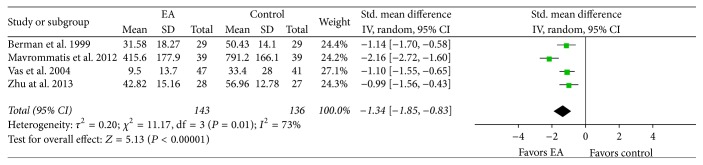
Effects of EA treatment versus control group interventions on WOMAC total scores. EA: electroacupuncture; WOMAC: Western Ontario and McMaster Universities Osteoarthritis Index.

*Comprehensive Outcomes of Knee OA Symptoms and Knee Joint Functions.* A meta-analysis was conducted using WOMAC total scores based on a scale designed to evaluate knee OA symptoms and dysfunction. In the meta-analysis of four studies [27, 34, 37, 45], in which 279 subjects participated, the EA treatment group showed more significant improvement in WOMAC total scores than the control group (SMD −1.34, CI 95% −1.85 to −0.83, and *I* ^2^ 73%; Figure 7).

*Quality of Life Outcomes*

**Figure 8:**
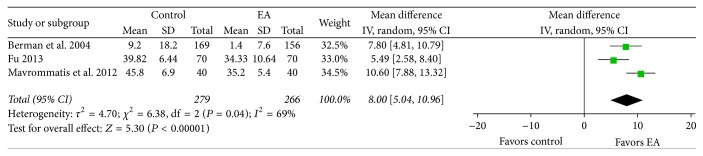
Effects of EA treatment versus control on the SF-36 physical scale. EA: electroacupuncture; SF-36: 36-item Short-Form Health Survey.

*Physical.* A meta-analysis was conducted using SF-36 physical scale data to evaluate physical state-related QOL outcomes. One-scale data was analyzed using the mean difference (MD). The MD was used to conduct the meta-analysis, thus analyzing data from a single study [32] reporting on change scores. The EA treatment group showed more significant improvement in physical state-related QOL than the control group (MD 8.00, CI 95% 5.04 to 10.96, and *I* ^2^ 69%; Figure 8).

**Figure 9:**
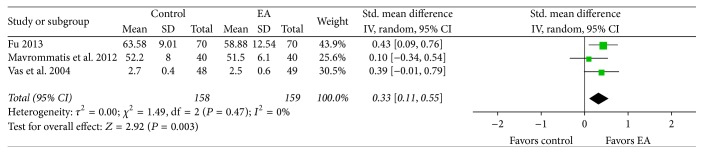
Effects of EA treatment versus control on mental state-related QOL. EA: electroacupuncture; QOL: quality of life.

*Mental.* At an end-point of more than four weeks following treatment, the EA treatment group showed more significant improvement in mental state-related QOL than the control group (SMD 0.33, CI 95% 0.11 to 0.55, and *I* ^2^ 0%; Figure 9).

## 5. Discussion

In the results of this study, the EA treatment group showed a significant reduction in pain due to knee OA in comparison to the control group. In addition, there was a significant improvement in comprehensive evaluation of knee OA and QOL among participants in the EA treatment group compared with the control group. Findings from the subgroup analysis of the control group show that the EA treatment plus drug therapy group experienced significantly reduced pain due to knee OA in comparison to the drug therapy alone group. Additionally, the EA treatment group showed significantly reduced pain due to knee OA in comparison to the sham EA group.

After excluding the results of meta-analysis on the mental state-related QOL of participants, there was a high measurement of heterogeneity among the other articles. There was no discernible lessening of this heterogeneity after the control group interventions were made uniform. This may indicate that other factors play a role in heterogeneity, with the exception of different types of interventions in the control group. One contributing factor to heterogeneity in the meta-analysis of this study involves the differences in EA interventions in the experimental group. Contributing factors to heterogeneity in acupuncture treatments include the location and number of acupuncture points, the proportional percentages of combined local and distal acupuncture points, individualized acupuncture points according to the diagnosis of a patient, and types of manual stimulation. Another variable, EA frequency, contributed to increasing the heterogeneity found in articles. No single study included in the meta-analysis of this study used the same acupuncture points for EA, while only two articles [32, 45] showed the same acupuncture points in EA treatments. The same frequency of 6 Hz for EA was found in only two articles [31, 33]. Other studies used different frequencies to conduct EA treatments, as well as different modes of electrical stimulation. Four articles [27, 34, 37, 45] featured alternately modified EA frequencies, three articles [31–33] used constant EA frequency, and one article [43] included no specification of EA frequency. Accordingly, the electrical feature of EA intervention may contribute to increasing the heterogeneity of meta-analysis of the current systematic review.

Further research should account for the electrical characteristics of EA treatment. In order to improve the function and symptoms of patients with knee OA, EA frequencies can be set differently to stimulate either motor systems for rehabilitation [58] or sensory systems for pain control [59]. Additionally, alternately modified EA frequency might be more advisable than application of a constant EA frequency [60].

Other factors generating considerable heterogeneity include the total number and frequency of EA treatment sessions, duration of treatments, and evaluation time points. There are no standards for clinically effective duration of EA treatment or evaluation time points [16]. Nevertheless, previous studies [16, 61] have conducted meta-analyses using data evaluated over certain evaluation time points to reduce heterogeneity and to verify clinically significant treatment effects on knee OA. Thus, meta-analysis in this paper was conducted on studies involving observation periods longer than five weeks based on the judgment of clinical experts in the field.

According to a recent Cochrane review [16], in order not to dilute the effects of acupuncture, continuing treatment is recommended to evaluate the prolonged effects. Further research should not allow a large time difference between the follow-up evaluation time point and the end-point of treatment. Accordingly, treatment is recommended to be maintained by tapered acupuncture treatments during the follow-up evaluation period [62].

There are substantial limitations in RCTs utilizing control group interventions such as drug therapy, education, or physiotherapy, because this methodology cannot exclude placebo effects [16]. When carefully designed, sham EA is one of the most important elements for excluding placebo effects and for focusing on the true effects of EA. The design of sham acupuncture, however, is as difficult as manipulating patient sensations, insofar as the “de-qi” sensation serves as one of the most significant contributors to a patient's perceptions of the effects of acupuncture [63]. Moreover, the mechanisms of acupuncture are mixed [64] and not clearly established [65]. A participant expects to experience a particular sensation from the sharp-needle form of acupuncture and electrical stimulation. This expectation makes it difficult to blind a participant. Eight studies [27, 29–32, 34, 36, 38] using sham EA treatments as control groups in RCTs were included in the current systematic review. Regarding the designs of these sham treatments, two articles [29, 30] conducted sham treatments with penetrating acupuncture applied on nonacupoints (off the meridian system), while four other articles [27, 31, 32, 34] conducted sham treatments with nonpenetrating acupuncture on the same acupoints as in the real treatment group. The remaining two articles [36, 38] conducted sham treatments with the attachment of patch-type electrodes without acupuncture onto the same acupoints as in the real treatment group. In five [30–32, 36, 38] of these articles, a light or sound to indicate operation of the electrical stimulator was used in order to distract participants in the process of mock electrical stimulation.

A design for sham treatment based on penetrating acupuncture is not desirable because the strategy may unwittingly cause activation of nonspecific mechanisms of acupuncture effects, which have nothing to do with stimulation of specific acupoints [66–68]. Sham treatments using patch-type electrodes instead of acupuncture may increase the possibility that participants cannot be properly blinded, given that they are able to see electrodes. The conclusion is that there are four articles [27, 31, 32, 34] with a suitable design for sham EA treatment in patients with knee OA. Of these, the two studies that utilized lights or sounds during the process [31, 32] represent the most suitable intervention design for credible sham EA treatment for knee OA in terms of patient perceptions. These two studies displayed low risk in the domains of random sequence generation and allocation concealment for risk of bias assessment. In the article by Jubb et al. [31], the EA treatment group showed significant improvement in pain due to knee OA in comparison to the sham treatment group, and the EA treatment group continued to show significant effects of pain relief in follow-up evaluations. The EA treatment group in Berman et al.'s article [32] showed more significant improvement in joint dysfunction due to knee OA in comparison to the sham treatment group at evaluation time points of eight weeks and 26 weeks. At 26 weeks of evaluation, the EA treatment group showed a significant decline in pain due to knee OA. Even in meta-analysis (Figure 5), the two articles [31, 32] showed a significant decline in pain due to knee OA in the EA treatment group in comparison to the sham treatment group.

Seven RCTs [26, 39, 41, 43, 46–48] were designed to compare EA treatment groups and MA treatment groups, five of which used the same acupuncture points for both treatment groups [26, 39, 43, 47, 48]. The other two RCTs [41, 46] used almost the same acupuncture points for the treatment groups, with the exception of four additional acupuncture points. According to the criteria of this review, data from studies [39, 46–48] that exclusively reported efficacy rate outcomes and data from studies [26, 41] with improper observation durations were not included in our meta-analysis. There were three articles [26, 41, 43] reporting outcomes that conformed to this criteria. In Plaster et al.'s article [26], immediate treatment effects experienced by both EA and MA treatment groups after one treatment session did not demonstrate any significant differences between the groups. In Fu's article [43], the EA treatment group demonstrated no significant differences in WOMAC or SF-36 scale score in comparison to the MA treatment group at four weeks, but there was a significant improvement at nine weeks compared to the MA treatment group. In Li's article [41], the MA treatment group at both 23 days and six months of evaluation showed more significant improvement in WOMAC and SF-36 scale scores than the EA treatment group. The MA treatment used in Li's article [41] is based on a technique of tug-of-war, which applies strong stimulation to acupuncture points by moving a needle in and out. This manual stimulation technique is not exactly comparable to the basic de-qi sensation that occurs for participants in other RCTs included in this review. Therefore, it is difficult to draw any conclusions on the comparison between EA and MA treatments for knee OA due to differences in evaluation time points and the unique technique of manual stimulation in the article by Li [41].

Although emerging studies are beginning to note that EA and MA treatments use different types of stimulations with different physiological effects, the existing research uses the term acupuncture for both MA and EA treatments without a clear distinction between the types of stimulation [21, 62]. One recent study [21] concluded that it is difficult to consider MA and EA treatments as interchangeable based on data regarding the physiological effects, clinical trials, and systematic reviews of existing studies. Because electrical stimulation may cause depolarization of surrounding tissues [21] and because manual stimulation causes mechanical transduction [69], the accompanying effects due to differences in types of stimulation are bound to be different. Additionally, MA and EA treatments cause activation of different areas in the central nervous system [70, 71]. By adjusting the frequency of applied EA, EA treatment can facilitate the release of particular neuropeptides from the central nervous system, subsequently activating self-healing mechanisms [72, 73]. These unique physiological mechanisms bolster the argument that EA treatments should be differentiated from MA treatments. In fact, the ACR guidelines [8] now conditionally recommend acupuncture treatments for OA based on the treatment effects, despite the publication of some studies [9, 16–20] demonstrating controversial results. The sensitivity analysis of a recent Cochrane review [16] concluded that EA treatments may reduce pain due to knee OA more significantly than MA treatment in patients, indicating a need for further study on this topic. Some recent articles [62, 74] were supportive of further research on the use of EA treatments for knee OA based on this current trend of study. However, the latest systematic review study [19] on the effects of acupuncture for knee excluded RCTs that utilized EA treatments and instead included RCTs that exclusively utilized MA treatments in order to reduce heterogeneity. One recent systematic review study [17], which included the effects of EA, used the term acupuncture to describe a blend of EA and MA treatments for meta-analysis. No systematic review study has been conducted to verify the effect of EA treatments for knee OA. The conclusion is that there is a need for a systematic review of RCTs utilizing EA treatment for knee OA. Indeed, a systematic review study of RCTs (including the Chinese CNKI database, which is one of the world's largest RCT databases) should be conducted. The current article serves to provide the foundation for further advanced studies by systematically reviewing RCTs published to date on the use of EA treatment for patients with knee OA.

*Quality of Evidence.* All studies included are RCTs. Most studies involved in the meta-analysis were assessed as low risk of bias. The quality of evidence was downgraded by one level with regard to inconsistency of results because *I* ^2^ value was between 50% and 75% in most meta-analyses. All studies directly compared the intervention, so there was no risk of the indirectness of evidence. Most studies had a sufficient sample size; therefore, the quality of evidence was not rated down due to imprecision.

We are moderately confident in the effect estimate in improvement with pain, comprehensive outcomes for knee OA, and QOL. The true effect would be close to the estimate of the effect, but it is possible to be considerably different.

*Limitation.* A limitation of this article is that its largest portion of included RCTs was shown to have a high risk of bias, and considerable heterogeneity was shown in the results of the meta-analysis. To conduct meaningful studies on the effects of EA for the treatment of knee OA, the study must consider the number of acupuncture points for EA, the location of acupuncture points on which electrical stimulation is applied, the frequency of electrical stimulation, criteria for the duration of treatment, guidelines for evaluation time points, and well-designed sham treatments for purposes of comparison.

## 6. Conclusion

This systematic review includes 31 RCTs, enrolling a total of 3,187 participants. Eight RCTs enrolling a total of 1,220 patients were included in a meta-analysis. The following conclusions were made based on the results of the systematic review and meta-analysis.

EA treatment can more significantly relieve the pain of patients with knee OA than control interventions and sham EA treatment.

EA treatment can more significantly improve comprehensive aspects of knee OA symptoms and knee joint functioning than control interventions.

EA treatment can more significantly improve the quality of life of patients with knee OA than control interventions.

Both the general features of electrical stimulation and the specific characteristics of EA acupuncture regimens should be considered for further study design and actual clinical practice using EA treatments in patients with knee OA.

## Figures and Tables

**Table 1:** Characteristics of included studies.

First author (year)	Sample size (M/F)	Diagnostic criteria (radiologic evidence)	Mean age	Experimental group				Control group		Outcomes
				Intervention type	Total session	Duration/evaluation time points after baseline	*n*	Intervention type	*n*	
Li [75] (2015)	76 (36/40)	Chinese Rheumatology Association criteria	n.r.	EA	15	3 weeks/3 weeks	38	Medication (celecoxib 100 mg bid)	38	HSS knee score

Wu [76] (2015)	100 (36/64)	ACR	61	EA	14	4 weeks/2 weeks, 4 weeks	50	Medication (topical diclofenac diethylamine emulgel 20 g qd)	50	Lequesne index VAS Rehabilitation value (peak torque of muscle, fatigue index)

Ying [39] (2015)	171 (76/95)	n.r.	58	EA	21	8 weeks/8 weeks	57	MA (same points)	57	Efficacy rate

Huang [40] (2014)	168 (85/83)	ACR	59.6	EA Medication (glucosamine hydrochloride 75 mg bid)	36	6 weeks/3 weeks, 6 weeks, 6 months	58	Medication (glucosamine hydrochloride 75 mg bid)/EA (same points)^*∗*^	53	Lequesne index Efficacy rate

Li [41] (2014)	100 (36/64)	ACR (K-L grade)	61	EA Physiotherapy (thermotherapy)	20	23 days/23 days, 6 months	50	MA (tug-of-war needling, similar points) physiotherapy (thermotherapy)	50	WOMAC VAS Efficacy rate

Plaster [26] (2014)	60 (11/49)	ACR	63.2	EA	1	1 day/immediately after treatment	30	MA (same points)	30	NRS TUG test Muscle strength PPT

Ruan [77] (2014)	72 (39/33)	ACR	55	EA	21	25 days/25 days	38	Medication (glucosamine hydrochloride 750 mg bid)	34	Lequesne index IEMG and MF values Efficacy rate

Ruan [42] (2014)	88 (42/46)	ACR	53.6	EA Medication (glucosamine hydrochloride 240 mg tid)	24	8 weeks/8 weeks	45	Medication (glucosamine hydrochloride 240 mg tid)	43	Efficacy rate

Fu [43] (2013)	208 (103/105)	ACR	58.3	EA	24	4 weeks/4 weeks, 9 weeks	70	Medication (ibuprofen 0.3 g bid)/MA (same points)^*∗*^	70	WOMAC SF-36 Efficacy rate

Zhao [44] (2013)	93 (n.r.)	ACR	n.r.	EA	12	4 weeks/1 week, 2 weeks, 4 weeks	33	Medication (celecoxib 20 mg tid)	19	VAS Knee function score (converted from IAC Lennox test and Lysholm scale)

Zhu [45] (2013)	55 (22/33)	ACR (K-L grade)	68.7	EA	14	4 weeks/4 weeks, 12 weeks	28	Medication (diclofenac sodium 75 mg qd)	27	WOMAC VAS Serum MMP-3 Efficacy rate

Che [78] (2012)	63 (25/38)	ACR	52	EA	10	20 days/20 days	32	Medication (diclofenac sodium 75 mg qd)	31	Efficacy rate

Li [46] (2012)	100 (37/63)	Chinese Orthopedic Association Criteria	53.8	EA	20	4 weeks/4 weeks	50	MA (similar points)	50	Efficacy rate

Mavrommatis [27] (2012)	120 (29/91)	ACR (K-L grade)	61.8	EA Medication (etoricoxib 60 mg qd)	16	8 weeks/4 weeks, 8 weeks, 12 weeks	40	Sham EA^†^ medication (etoricoxib 60 mg qd)/medication (etoricoxib 60 mg qd)^*∗*^	40	WOMAC VAS SF-36v2 PPT

Teng [47] (2012)	86 (32/54)	ACR	61	EA	24	8 weeks/8 weeks	30	MA (same points)	26	Efficacy rate

Ji [79] (2011)	70 (31/39)	ACR	56	EA	24	8 weeks/8 weeks	35	Medication (meloxicam 7.5 mg qd)	35	Lequesne index VAS ISOA IDS

Meng [28] (2011)	69 (16/53)	n.r.	n.r.	EA Medication (acetaminophen 500 mg qid/free) Physiotherapy (n.r.)	9	3 weeks/8 weeks, 20 weeks	28	Medication (acetaminophen 500 mg qid/free) Physiotherapy (n.r.)	18	Knee score and functional score (Knee Society Clinical Rating System as modified by Insall) VAS Medication dose

Lu [29] (2010)	20 (n.r.)	n.r. (K-L grade)	63.8	EA	1	1 day/immediately after treatment	10	Sham EA^§^	10	VAS Gait analysis

Wu [80]. (2010)	80 (34/46)	ACR	48.6	EA	20	23 days/23 days	40	Medication (fenbid 30 mg bid)	40	Efficacy rate

Ahsin [30] (2009)	84 (n.r.)	ACR	n.r.	EA	10	10 days/10 days	26	Sham EA^§^	58	VAS WOMAC Plasma *β*-endorphin cortisol

Hou [48] (2008)	50 (24/26)	ACR	62.5	EA	24	30 days/30 days	25	MA (same points)	25	Efficacy rate

Jubb [31] (2008)	68 (13/55)	n.r.	65.1	EA	10	5 weeks/5 weeks, 9 weeks	34	Sham EA^†^	34	WOMAC VAS Euro-Qol score Plasma *β*-endorphin

Wu [49] (2008)	40 (15/25)	ACR	61.8	EA	12	4 weeks/4 weeks	20	Medication (diclofenac sodium 25 mg tid)	20	Lysholm scale

Qiu [53] (2006)	60 (9/51)	ACR	55.7	EA	8	4 weeks/2 weeks, 4 weeks	30	Medication (Voltaren 75 mg qd)	30	WOMAC

Berman [32] (2004)	570 (205/365)	n.r. (K-L grade)	65.5	EA	23	26 weeks/4 weeks, 8 weeks, 14 weeks, 26 weeks	190	Sham EA^†^/education^*∗*^	191	WOMAC Patient global assessment Walk distance for 6 minutes SF-36

Tukmachi [33] (2004)	29 (5/24)	n.r. (K-L grade)	62	EA Medication (current medication, n.r.)	10	5 weeks/5 weeks, 9 weeks	10	Medication (current medication, n.r.)/EA (same points)^*∗*^	10	WOMAC VAS

Vas [34] (2004)	97 (16/81)	ACR	67.1	EA Medication (diclofenac 50 mg tid/free)	12	12 weeks/12 weeks	48	sham EA^†^ medication (diclofenac 50 mg tid/free)	49	WOMAC VAS PQLC Medication dose

Ng [35] (2003)	24 (1/23)	n.r.	85	EA	8	2 weeks/2 weeks, 4 weeks	8	General education	8	NRS TUG test Passive ROM

Sangdee [36] (2002)	193 (43/150)	ACR	62.9	EA Medication (diclofenac 25 mg tid/free)	12	4 weeks/4 weeks	49	Sham ES^‡^ medication (diclofenac 25 mg tid/free)/EA with placebo medication^*∗*^/sham ES^‡^ with placebo medication^*∗*^	49	WOMAC VAS Lequesne index Time to walk 50 feet Medication dose

Berman [37] (1999)	73 (28/45)	ACR (K-L grade)	65	EA	16	8 weeks/4 weeks, 8 weeks, 12 weeks	37	Medication (n.r.)	36	WOMACLequesne index

Yurtkuran [38] (1999)	100 (9/91)	n.r.	58.1	EA	10	2 weeks/2 weeks	25	Sham ES^‡^	25	PPI Stiffness Time to walk 50 feet Muscle strength (quadriceps) Active knee flexion

M/F: number of males/number of females, *n*: number, ACR: American College of Rheumatology criteria, K-L grade: Kellgren-Lawrence grade, n.r.: not reported, EA: electroacupuncture, MA: manual acupuncture, ES: electrical stimulation, qd: once a day, bid: twice a day, tid: three times a day, qid: four times a day, WOMAC: Western Ontario and McMaster Universities Osteoarthritis Index, VAS: visual analogue scale, NRS: numeric rating scale, PQLC: profile of quality of life in the chronically ill, PPI: present pain intensity, PPT: pressure pain threshold, TUG test: Timed Up-and-Go test, SF-36v2: Short-Form 36 version 2, SF-36: Short-Form 36-item Health Survey, HSS: Hospital for Special Surgery, IEMG and MF values: integrated electromyogram and median frequencies values measured by surface electromyography, ISOA: index of severity of osteoarthritis, and IDS: index of disease severity.

^*∗*^Multiple-arm study.

^†^Sham electroacupuncture using nonpenetrating acupuncture with sham electrical stimulation at the same points.

^‡^Sham electrical stimulation without acupuncture using patch electrodes at the same points.

^§^Sham electroacupuncture using penetrating acupuncture with sham electrical stimulation at nonacupoints.

**Table 2:** Summary of information related to electroacupuncture treatments.

First author (year)	Acupuncture rationale	Details of needling					Practitioner background
		Acupuncture points	ES frequency, Duration	De-qi	Depth of insertion	Needle type (diameter/length)	
Li [75] (2015)	TCM	EX-LE5, SP10, ST36, ST34, GB34, SP9, Ashi point EA points: 4 points among above acupoints (n.r.)	20 Hz, 30 min	Elicited	n.r.	n.r./50.0 mm	n.r.

Wu [76] (2015)	TCM	EX-LE5 EA points: all acupoints	40 Hz, 45~60 min	n.r.	33.3 mm	n.r./50.0 mm	n.r.

Ying [39] (2015)	TCM	ST35, EX-LE4, EX-LE2, SP10, ST36, GB34, SP9, BL40 EA points: all acupoints	n.r., 30 min	Elicited	25.4~38.1 mm	0.30 mm/40 mm	n.r.

Huang [40] (2014)	TCM	Ashi point, ST35, EX-LE4, SP10, EX-LE2, SP6, SP9, ST36, LR3 EA points: 4 points among above acupoints (n.r.)	15 Hz, 30 min	Elicited	15~20 mm	0.35 mm/25 mm	n.r.

Li [41] (2014)	TCM	ST35, EX-LE4, GB34, SP9, GB33, ST34, SP10, Ashi point EA points: ST35, EX-LE4, GB34, SP9, GB33, Ashi point	2 Hz, 30 min	Elicited	25~40 mm	0.35 mm/40 or 50 mm	n.r.

Plaster [26] (2014)	TCM according to previous study [31]	LI4, LR3, ST36, ST35, EX-LE5, SP10 EA points: all acupoints	3 Hz and 100 Hz (alternate), 30 min	n.r.	5 mm	0.25 mm/30 mm	n.r.

Ruan [77] (2014)	TCM	EX-LE4, SP9, LR8, SP10, ST35, ST36, GB33, ST34, EX-LE2, ST32, Ashi point EA points: SP9, SP10, ST36, ST34, EX-LE2, ST32	n.r., 30 min	Elicited	33.3~5.0 mm	0.30 mm/50 mm	n.r.

Ruan [42] (2014)	TCM	ST34, SP10, GB34, ST36, SP9, GB39, Ashi point EA points: all acupoints	n.r., 30 min	Elicited	n.r.	n.r./50.0 mm	n.r.

Fu [43] (2013)	TCM	EX-LE5, SP9, GB34, BL40, EX-LE2 Individualized acupoints: BL23, CV3, SP10, BL57, BL23, BL25, ST36, SP6, BL23, KI3, SP8, ST40 EA points: EX-LE5, SP9, GB34	n.r., 30 min	Elicited	n.r.	0.35 mm/50~60 mm	n.r.

Zhao [44] (2013)	TCM	CV8, EX-LE5, SP10, ST34 EA points: SP10, EX-LE5, ST34	n.r., 30 min	Elicited	n.r.	0.25 mm/40~70 mm	n.r.

Zhu [45] (2013)	TCM	Xian, GB34, SP9, ST36, Ashi point EA points: Xian	2 Hz and 100 Hz (alternate), 20 min	Elicited	n.r.	0.25 mm/40 mm	n.r.

Che [78] (2012)	TCM, western medical	EX-LE4, ST35 EA points: all acupoints	2 Hz, 45 min	Elicited	60 mm	0.45 mm/75 mm	n.r.

Li [46] (2012)	TCM	EX-LE5, Ashi point, ST34, SP10, GB34, SP9, ST36, GB39 Proximal acupuncture points: EX-LE5, Ashi point EA points: EX-LE5	n.r., 30 min	Elicited	33.3 mm	0.25 mm/40 mm	n.r.

Mavrommatis [27] (2012)	TCM	ST36, SP9, SP10, GB34, Ex-LE 2, Ex-LE5, LI4, KI3, ST40, SP6 EA points: ST36, SP9, GB34, SP10	2 Hz~6 Hz, 20 min	Elicited	n.r.	0.25 mm/30 mm	Doctor specializing in acupuncture^*∗*^

Teng [47] (2012)	TMC	ST35, EX-LE4, EX-LE2, SP10, ST36, GB34 EA points: all acupoints	n.r., 30 min	Elicited	25.4~38.1 mm	0.30 mm/40 mm	n.r.

Ji [79] (2011)	TMC	ST35, EX-LE4, GB33, BL40, ST36, GB34, GB39 Individualized acupoints: SP10, BL17, BL23, CV4, SP9, SP6, BL11, BL23 EA points: ST35, EX-LE4	40 Hz~60 Hz, 30 min	Elicited	n.r.	0.30 mm/40 mm	n.r.

Meng [28] (2011)	n.r.	n.r.	n.r.	n.r.	n.r.	n.r.	n.r.

Lu [29] (2010)	TCM	GB34, SP9, SP10, SP34, ST36 EA points: all acupoints	2 Hz, 30 min	Elicited	10~15 mm	n.r.	Experienced acupuncturist

Wu [80]. (2010)	TCM, western medical	EX-LE5, SP10, ST34 EA points: all acupoints	n.r., 30 min	Elicited	26.7~40.0 mm	0.35 mm/50 mm	n.r.

Ahsin [30] (2009)	TCM	ST34, ST35, ST36, LR8, SP10, ST44 EA points: all acupoints	3 Hz, 20~25 min	Elicited	10~30 mm	n.r./30 mm	Qualified acupuncturist

Hou [48] (2008)	TCM	ST35, EX-LE2, ST34, GB34, SP9 EA points: all acupoints	3 Hz, 30 min	Elicited	n.r.	0.30 mm/50 mm	n.r.

Jubb [31] (2008)	TCM	LI4, SP10, EX-LE5, SP9, GB34, ST36, LR3, BL40, BL57 EA points: EX-LE5, SP9, GB34, BL40 BL57	6 Hz, 20 min	Elicited	10~15 mm	0.25 mm/30 mm	Acupuncturist

Wu [49] (2008)	TCM	EX-LE5, EX-LE2, SP10, SP11, ST34, ST36, SP9 EA points: EX-LE5, SP10, SP11	n.r., 20 min	Elicited	n.r.	n.r./n.r.	n.r.

Qiu [53] (2006)	TCM, western medical	EX-LE5 EA points: all acupoints	2 Hz and 100 Hz (alternate), 30 min	Elicited	5.1~6.4 mm	0.45 mm/75 mm	n.r.

Berman [32] (2004)	TCM	GB34, SP9, ST36, ST35, EX-LE5, UB60, GB39, SP6, KI3 EA points: EX-LE5	8 Hz, 20 min	Elicited	7.6~25.4 mm	0.25 mm/38.1 or 25.4 mm	State-licensed acupuncturists who have at least 2 years of clinical experience

Tukmachi [33] (2004)	TCM	LI4, SP10, EX-LE5, SP9, GB34, ST36, LR3, BL40, BL57 EA points: EX-LE5, SP9, GB34, BL40, BL57	6 Hz, 20 min	Elicited	10~15 mm	0.25 mm/30 mm	Acupuncturist

Vas [34] (2004)	TCM	GB34, SP9, Xian, ST36, LI4 Individualized acupoints: KI3, SP6, ST40 EA points: GB34, SP9, Xian, ST36	2 Hz and 15 Hz (alternate), 20 min	Elicited	n.r.	0.25 mm/45 mm	Doctor specializing in acupuncture^†^

Ng [35] (2003)	TCM according to previous study [81]	ST35, EX-LE4 EA points: all acupoints	2 Hz, 20 min	Elicited	10~15 mm	0.25 mm/40 mm	n.r.

Sangdee [36] (2002)	TCM	ST35, EX-LE4, LR8, Trigger point (at the level of the joint line, midpoint between EX-LE4 and LR8) EA points: all acupoints	2 Hz, 20 min	Not intended	Superficially (under 12.7 mm)	n.r.	Acupuncturist who received acupuncture training in the People's Republic of China

Berman [37] (1999)	TCM	GB34, SP9, ST36, ST35, EX-LE5, UB60, GB39, SP6, KI3 EA points: ST35, EX-LE5	2.5 Hz~4 Hz, 20 min	Elicited	10.2–15.2 mm	0.22 mm/25.4 mm	n.r.

Yurtkuran [38] (1999)	TCM	SP9, GB34, ST34, ST35 EA points: all acupoints	4 Hz, 20 min	n.r.	12.7–25.4 mm	0.25 mm/40 mm	n.r.

TCM: traditional Chinese medicine, EA: electroacupuncture, ES: electrical stimulation, Hz: hertz, min: minutes, and n.r.: not reported.

^*∗*^Accredited by the International Council of Medical Acupuncture and Related Techniques (ICMART) and the Medical Acupuncture Society of Northern Greece.

^†^Accredited by Beijing University of Medical Sciences (China) and by the Scientific Society of Medical Acupuncture.

## Appendix

Search strategies for MEDLINE/PubMed databases are as follows:

(1) osteoarthritis [MeSH]
(2) osteoarthritis [tw]
(3) “osteo arthriti^*∗*^” [tw]
(4) “degenerative arthritis” [tw]
(5) arthritis [tw]
(6) osteoarthrosis [tw]
(7) “osteo arthros^*∗*^” [tw]
(8) arthrosis [tw]
(9) osteoarthritides [tw]
(10) osteoarthroses [tw]
(11) arthroses [tw]
(12) OA [tw]
(13) osteo^*∗*^ [tw]
(14) gonarthrosis [tw]
(15) gonarthritis [tw]
(16) knee [tw]
(17) “knee pain” [tw]
(18) arthralgia [tw]
(19) 1 or 2 or 3 or 4 or 5 or 6 or 7 or 8 or 9 or 10 or 11 or 12 or 13 or 14 or 15 or 16 or 17 or 18
(20) acupuncture [MeSH]
(21) acupuncture [tw]
(22) “acupuncture therapy” [MeSH]
(23) “acupuncture therapy” [tw]
(24) electroacupuncture [MeSH]
(25) electroacupuncture [tw]
(26) “electroaucpuncture therapy” [MeSH]
(27) “electroacupuncture therapy” [tw]
(28) “electric acupuncture” [tw]
(29) “electrical acupuncture” [tw]
(30) electro-acupuncture [tw]
(31) “electro acupuncture” [tw]
(32) “electrical stimulation therapy” [MeSH]
(33) “electrical stimulation therapy” [tw]
(34) “transcutaneous electric nerve stimulation” [MeSH]
(35) “transcutaneous electric nerve stimulation” [tw]
(36) TENS [tw]
(37) 20 or 21 or 22 or 23 or 24 or 25 or 26 or 27 or 28 or 29 or 30 or 31 or 32 or 33 or 34 or 35 or 36
(38) “randomised controlled trial” [Publication Type]
(39) “randomised controlled trials as topic” [MeSH]
(40) “random allocation” [MeSH]
(41) “double-blind method” [MeSH]
(42) “single-blind method” [MeSH]
(43) placebo [MeSH]
(44) random^*∗*^ [tw]
(45) rct [tw]
(46) rct's [tw]
(47) rcts [tw]
(48) placebo^*∗*^ [tw]
(49) 38 or 39 or 40 or 41 or 42 or 43 or 44 or 45 or 46 or 47 or 48
(50) 19 and 37 and 49
